# Genome-Wide Linkage Mapping of QTL for Yield Components, Plant Height and Yield-Related Physiological Traits in the Chinese Wheat Cross Zhou 8425B/Chinese Spring

**DOI:** 10.3389/fpls.2015.01099

**Published:** 2015-12-18

**Authors:** Fengmei Gao, Weie Wen, Jindong Liu, Awais Rasheed, Guihong Yin, Xianchun Xia, Xiaoxia Wu, Zhonghu He

**Affiliations:** ^1^Key Laboratory of Soybean Biology, Soybean Research Institute, Ministry of Education, Northeast Agricultural UniversityHarbin, China; ^2^National Wheat Improvement Center, Institute of Crop Science, Chinese Academy of Agricultural SciencesBeijing, China; ^3^Keshan Sub-Academy, Heilongjiang Academy of Agricultural SciencesKeshan, China; ^4^International Maize and Wheat Improvement Center (CIMMYT) China Office, c/o Chinese Academy of Agricultural SciencesBeijing, China; ^5^Zhoukou Academy of Agricultural SciencesZhoukou, China

**Keywords:** linkage analysis, molecular marker, QTL, *Triticum aestivum*, wheat 90K SNP array

## Abstract

Identification of genes for yield components, plant height (PH), and yield-related physiological traits and tightly linked molecular markers is of great importance in marker-assisted selection (MAS) in wheat breeding. In the present study, 246 F_8_ RILs derived from the cross of Zhou 8425B/Chinese Spring were genotyped using the high-density Illumina iSelect 90K single nucleotide polymorphism (SNP) assay. Field trials were conducted at Zhengzhou and Zhoukou of Henan Province, during the 2012–2013 and 2013–2014 cropping season under irrigated conditions, providing data for four environments. Analysis of variance (ANOVA) of agronomic and physiological traits revealed significant differences (*P* < 0.01) among RILs, environments, and RILs × environments interactions. Broad-sense heritabilities of all traits including thousand kernel weight (TKW), PH, spike length (SL), kernel number per spike (KNS), spike number/m^2^ (SN), normalized difference in vegetation index at anthesis (NDVI-A) and at 10 days post-anthesis (NDVI-10), SPAD value of chlorophyll content at anthesis (Chl-A) and at 10 days post-anthesis (Chl-10) ranged between 0.65 and 0.94. A linkage map spanning 3609.4 cM was constructed using 5636 polymorphic SNP markers, with an average chromosome length of 171.9 cM and marker density of 0.64 cM/marker. A total of 866 SNP markers were newly mapped to the hexaploid wheat linkage map. Eighty-six QTL for yield components, PH, and yield-related physiological traits were detected on 18 chromosomes except 1D, 5D, and 6D, explaining 2.3–33.2% of the phenotypic variance. Ten stable QTL were identified across four environments, viz. *QTKW.caas-6A.1, QTKW.caas-7AL, QKNS.caas-4AL, QSN.caas-1AL.1, QPH.caas-4BS.2, QPH.caas-4DS.1, QSL.caas-4AS, QSL.caas-4AL.1, QChl-A.caas-5AL*, and *QChl-10.caas-5BL*. Meanwhile, 10 QTL-rich regions were found on chromosome 1BS, 2AL (2), 3AL, 4AL (2), 4BS, 4DS, 5BL, and 7AL exhibiting pleiotropic effects. These QTL or QTL clusters are tightly linked to SNP markers, with genetic distances to the closest SNPs ranging from 0 to 1.5 cM, and could serve as target regions for fine mapping, candidate gene discovery, and MAS in wheat breeding.

## Introduction

Wheat (*Triticum aestivum* L.) is the third most important cereal food crop after maize (*Zea mays* L.) and rice (*Oryza sativa* L.; Green et al., 2012; Edae et al., 2014). It accounts for about 19% of total grain production among the principal cereal crops, and provides 55% of the carbohydrate consumed by the human population in the world (Gupta et al., 1999; Bagge et al., 2007). Food security is becoming a serious concern for the future due to a rapidly increasing population, the gradual decrease in arable land area, shortages of water and other input resources, and predicted climate change impacts on crop yield. Thus, it is very important to increase the yields of all food crops to avert predicted food security crises (Yang et al., 2012).

Wheat GY is a complex quantitative trait with components such as spike number (SN), kernel number per spike (KNS), and thousand kernel weight (TKW). Potential yield is closely associated with plant photosynthesis (Reynolds et al., 2011). Genetic improvement of yield components and physiological traits can certainly increase grain yield (GY). Quantitative trait loci (QTL) mapping is a key approach for understanding the genetic architecture of yield components and physiological traits in wheat (Holland, 2007). Previously, QTL mapping using various segregating populations was conducted for plant height (PH), spike length (SL), SN, KNS, and TKW (Börner et al., 2002; Kumar et al., 2007; Cuthbert et al., 2008; Golabadi et al., 2011; Bennett et al., 2012). However, QTL were defined by relatively large genetic distances due to the limited numbers of markers. In addition, QTL for physiological traits were rarely reported, except a few association studies for SPAD value of chlorophyll content (Chl), normalized difference in vegetation index (NDVI), and canopy temperature (CT) in spring wheat (Edae et al., 2014; Pinto and Reynolds, 2015; Sukumaran et al., 2015).

The recently developed high-density single nucleotide polymorphism (SNP) gene-chip technology provides a superior approach for QTL mapping, because SNP markers have less errors in evaluation, higher accuracy and particularly higher numbers than SSR markers (Birkhead et al., 2010; Yu et al., 2011). In addition, SNPs can be employed to survey the structure and progressive history of populations, as a tool for association and linkage mapping to detect QTL and to build high-density linkage maps (Aranzana et al., 2005; Akhunov et al., 2009). During the past 5 years, high-density SNP data were increasingly used to identity QTL in bi-parental populations and genome-wide association studies (GWAS) in important crops and animals (Rafalski, 2002; Tian et al., 2011; Zhao et al., 2011; Cook et al., 2012; Jia et al., 2013) due to their high call frequency, locus specific, co-dominant inheritance, simple documentation, potential for analysis, and low error rates (Gupta et al., 1999; Schlotterer, 2004). Many QTL for agronomic and quality traits have been successfully identified in maize and rice using GWAS and high-throughput SNP genotyping (Huang et al., 2010, 2011; Li et al., 2012; Yang et al., 2013). QTL mapping using segregating populations and SNP chip technology has been reported in pea, potato, watermelon, and barley (Ariyadasa et al., 2014; Lambel et al., 2014; Prashar et al., 2014; Sindhu et al., 2014). During the last 2 years, several QTL and association mapping studies were conducted for disease resistance, pre-harvest sprouting, and yield related traits using 9K and 90K SNP chips in wheat (Cabral et al., 2014; Sela et al., 2014; Wang et al., 2014; Sukumaran et al., 2015).

Zhou 8425B, an elite Chinese wheat line developed by the Zhoukou Academy of Agricultural Sciences in 1984, has a semi-dwarf PH, large spike, high TKW and multiple disease resistance (Li et al., 2006; Zhao et al., 2008; Xiao et al., 2011). More than 100 cultivars derived from this line have been grown on an accumulated area of over 33 million ha in China during the past 20 years (Yin et al., 2009). Currently, more than half of the wheat cultivars in Henan province, the largest wheat production region in China, are derivatives of Zhou 8425B. Therefore, it should be interesting to dissect the genetic components of this elite line for more efficient use in breeding programs. The present study used a 90K Infinium iSelect SNP assay to screen 246 RILs from the cross of Zhou 8425B/Chinese Spring; 5636 polymorphic SNPs were used to generate a high-density chromosome linkage map. The objectives were to identify QTL for yield components, PH, and yield-related physiological traits, and their tightly linked SNP markers for marker-assisted selection (MAS) in wheat breeding.

## Materials and methods

### Plant materials and field trials

A total of 246 F_8_ RILs derived from the cross of Zhou 8425B/Chinese Spring were used in this study. Field trials were performed at Zhengzhou and Zhoukou of Henan Province, during the 2012–2013 and 2013–2014 cropping seasons, providing data for four environments. The RILs were planted in randomized complete blocks with three replicates at each location. Plots consisted of four 1.5 m rows with 20 cm between rows. Approximately 50 seeds were sown evenly in each row. The field trials were managed following the local normal practice.

### Phenotyping

The NDVI and Chl were measured at anthesis and 10 days post-anthesis in each plot. NDVI was measured by scanning plants with a portable spectroradiometer (GreenSeeker, Ntech Industries, Inc, Ukiah, CA). Chl was scored as the average of six flag leaves per plot using a chlorophyll meter SPAD-502 (Inolta, Japan). PH was measured from the ground to the tip of the spike excluding awns at the late grain-filling stage. SL was recorded as average values of five spikes per plot. SN was scored in a 1 m single row section and then transformed to SN per m^2^. KNS was calculated from the mean of 30 randomly selected spikes in each plot. After harvest, TKW was measured by weighing duplicates of 500 kernels from each plot. GY was determined as the weight of grain harvested per unit area (kg/m^2^).

### Phenotypic data analysis

The phenotypic data analyses were conducted with SAS v. 9.2 software (SAS Institute Inc, Cary, NC). PROC GLM was used in ANOVA, where genotypes were considered as fixed effects, and environments and replicates nested in environments were considered as random effects. Correlation analysis between parameters was performed using the “PROC CORR” procedure. Broad-sense heritability was estimated in all environments aqs $h_{B}^{2}=\sigma_{g}^{2}∕\left(\sigma_{g}^{2}+\sigma_{ge}^{2}∕r+\sigma_{\varepsilon }^{2}∕re\right)$, where the genetic variance $\sigma_{g}^{2}=\left(MS_{f}-MS_{fe}\right)∕re$, genotype × environment interaction variance $\sigma_{ge}^{2}=\left(MS_{fe}-MS_{e}\right)∕r$, error variance σ^2^_ε_ = *MS_e_*, *MS*_*f*_ = genotype mean square, *MS*_*fe*_ = genotype × environment interaction mean square, *MS*_*e*_ = error mean square, and *r* and *e* were the numbers of replicates and environments, respectively.

### SNP genotyping

The 246 RILs and their parents were genotyped with the 90K iSelect SNP array (Wang et al., 2014) from CapitalBio Corporation (Beijing, China; http://www.capitalbio.com). Genotypic clusters for every SNP were determined following the manual for Genome Studio version 1.9.4 with the polyploid clustering version 1.0.0 (Illumina; http://www.illumina.com), based on the data from all the genotypes. SNPs were filtered by excluding those with monomorphic or with poor quality data. SNP markers with missing parental genotype information, where the parental genotypes were inconsistent with progeny genotypic ratios were removed. SNPs with large numbers of missing values (20% or more) were not included in map construction. Molecular markers for dwarf genes *Rht-B1b* and *Rht-D1b* were used to confirm the association with PH.

### Linkage map construction

SNP markers were grouped using IciMapping 4.0 software (http://www.isbreeding.net). Linkage analysis was performed using JoinMap 4.0 (Stam, 1993). Then, the linkage map was constructed using MapChart 2.2 (http://www.earthatlas.mapchart.com). Map distances between markers were calculated with the Kosambi mapping function. Each linkage group was oriented from the short (S) to long (L) chromosome arms, and the position and the order of the markers were compared with wheat 90K consensus SNP map (Wang et al., 2014).

### QTL analysis

QTL analysis was performed using inclusive composite interval mapping (ICIM) with IciMapping 4.0 software (Li et al., 2007a). Phenotypic values of all lines in each environment, and the averaged phenotypic values from the four environments, were used for QTL detection. Missing phenotypic data were deleted using the “Deletion” command. The walking speed chosen for all QTL was 1.0 cM, with *P* = 0.001 in stepwise regression. Based on 2000 permutations at a probability level of 0.01, the LOD scores to declare significant QTL for all traits ranged from 2.0 to 2.5 across four environments, thus a LOD threshold of 2.5 was chosen for declaration of putative QTL. Each QTL was represented by a 20 cM interval with the LOD maximum as center. The phenotypic variance explained (PVE) was estimated through stepwise regression (Li et al., 2007a).

## Results

### Phenotypic evaluation

ANOVA were conducted for TKW, KNS, SN, PH, SL, Chl-A, Chl-10, NDVI-A, and NDVI-10 across four environments. There were significant differences among the 246 RILs for all traits. The frequency distributions of TKW, KNS, SN, PH, SL, Chl-A, Chl-10, NDVI-A, and NDVI-10 for the RILs in each environment were continuous (Figure S1), indicating polygenic control. Based on data averaged across four environments, TKW ranged from 26.5 to 52.6 g with an average of 37.2 g, KNS ranged between 41 and 74 with an average of 53, and SN ranged from 318 to 671 with an average of 465. PH and SL ranged from 60.6 to 125.9 cm and 6.9 to 16.0 cm, with averages of 100.9 and 10.2 cm, respectively. Chl-A and Chl-10 ranged between 38.6 and 76.5 units and between 28.7 and 58.1 units, with an average of 46.5 and 47.8 units, respectively. Similarly, NDVI-A and NDVI-10 ranged from 0.51 to 0.81 and 0.40 to 0.71 units, with averages of 0.74 and 0.55, respectively (Table S1). TKW, PH, SL, and NDVI-10 showed higher heritabilities, ranging from 0.88 to 0.94, followed by Chl-10 (0.85), Chl-A (0.79), KNS (0.78), SN (0.74), and NDVI-A (0.65). ANOVA of the nine traits revealed significant differences (*P* < 0.01) among RILs, environments, and genotype × environment interactions (Table 1), confirming strong environmental influences on these traits.

**Table 1:** **Analysis of variance for yield components, plant height, and physiological traits in F_8_RILs from Zhou 8425B/Chinese Spring across four environments**.

**Source of variance**	***df***	**Sum of squares**								
		**TKW**	**KNS**	**SN**	**PH**	**SL**	**Chl-A**	**Chl-10**	**NDVI-A**	**NDVI-10**
Environments	3	30,765^**^	4676^**^	6,570,684^**^	163,966^**^	1806^**^	9428^**^	5974^**^	13.8^**^	9.09^**^
Lines	245	57,359^**^	89,731^**^	1,035,994^**^	605,189^**^	3971^**^	19,547^**^	46,644^**^	3.6^**^	9.03^**^
Replicates	2	29	170	4596	128	201^**^	113^**^	574^**^	0.01^**^	0.02^**^
Lines × Environments	735	90,428^*^	51,955^**^	717,092^**^	96,937^**^	1051^**^	10,527^**^	17,795^**^	2.5^**^	3.08^**^
Error	1966	6607	54,938	108,3116	103,483	1090	8235	22,346	1.2	2
Broad-sense heritability		0.94	0.78	0.74	0.94	0.90	0.79	0.85	0.65	0.88

* *Significant at P = 0.05*,

** *Significant at P = 0.01*.

*TKW, thousand kernel weight; KNS, kernel number per spike; SN, spike number/m^2^; PH, plant height; SL, spike length; Chl-A, SPAD value of chlorophyll content at anthesis; Chl-10, SPAD value of chlorophyll content at 10 days post-anthesis; NDVI-A, normalized difference in vegetation index at anthesis; NDVI-10, normalized difference in vegetation index at 10 days post-anthesis*.

### Correlations between traits

Pearson's coefficients of correlation were calculated for all traits based on the data averaged over four environments (Table 2). KNS was positively correlated with SL (*r* = 0.43). The highest negative correlation was observed between TKW and SN (*r* = −0.36). KNS exhibited positive correlations with Chl-10 (*r* = 0.45) and NDVI-A (*r* = 0.38). SN was positively correlated with NDVI-A (*r* = 0.41) and NDVI-10 (*r* = 0.37). PH exhibited a negative correlation with Chl-A (*r* = −0.44). Chl-A was positively correlated with Chl-10 (*r* = 0.65). Chl-10 showed positive correlation with NDVI-A (*r* = 0.51) and NDVI-10 (*r* = 0.51). The maximum positive correlation was between NDVI-A and NDVI-10 (*r* = 0.78).

**Table 2:** **Pearson's coefficient of correlation for average of yield components, plant height, and physiological traits across four environments**.

**Trait**	**TKW**	**KNS**	**SN**	**PH**	**SL**	**Chl-A**	**Chl-10**	**NDVI-A**
KNS	−0.12							
SN	−0.36^**^	−0.25^**^						
PH	0.19^*^	−0.02	0.04					
SL	0.08	0.43^**^	−0.19^**^	−0.03				
Chl-A	0.24^**^	0.24^**^	−0.23^**^	−0.44^**^	0.09			
Chl-10	0.20^**^	0.45^***^	−0.13	−0.26^**^	0.21^**^	0.65^**^		
NDVI-A	−0.06	0.38^**^	0.41^**^	−0.06	0.24^**^	0.10	0.51^**^	
NDVI-10	−0.10	0.26^**^	0.37^**^	−0.11	0.14	0.01	0.51^**^	0.78^**^

* and ** *represent significance at P < 0.05 and P < 0.01, respectively*.

*TKW, thousand kernel weight; KNS, kernel number per spike; NS, number of spikes; PH, plant height; SL, spike length; Chl-A, SPAD value of chlorophyll content at anthesis; Chl-10, SPAD value of chlorophyll content at 10 days post-anthesis; NDVI-A, seedling normalized difference in vegetation index at anthesis; NDVI-10, seedling normalized difference in vegetation index at 10 days post-anthesis*.

### SNP genotyping

Among 81,587 SNPs used in screening the Zhou 8425B/Chinese Spring population, 7514 SNP markers (9.2%) were polymorphic between the two parental lines. Of those, 192 markers had more than 20% missing data points in the RILs, and 1686 were not anchored on the linkage map.

### Linkage map construction

Twenty-one linkage groups corresponding to the 21 hexaploid wheat chromosomes were constructed from the 5636 high-quality polymorphic SNP markers (Tables S2, S3); 2457 (43.6%) were localized to the A genome with a total length of 1668.1 cM and average marker density of 0.68 cM, 2838 (50.4%) were mapped to the B genome with a total length of 1276.8 cM and average marker density of 0.45 cM, and 341 were mapped to the D genome with a total length of 664.5 cM and average marker density of 1.95 cM. Ninety-four percent of markers mapped to the A and B genomes, indicating that SNP markers on those genomes were much more polymorphic than those in the D genome. All linkage maps covered 3609.4 cM with an average chromosome length of 171.9 cM, ranging from 21.0 cM (6D) to 303.7 cM (7A). The number of SNP markers in each wheat chromosome ranged from 10 mapped on chromosome 3D to 599 on chromosome 5B. The SNP markers were well distributed throughout the genome, although chromosomes 3D, 4D, and 7D exhibited lower marker densities. The overall SNP density was 0.64 cM, with the highest density of 0.28 cM on chromosome 2B, and the lowest density of 9.10 cM on chromosome 3D.

### QTL analysis of grain yield and related traits

**Figure 1:**
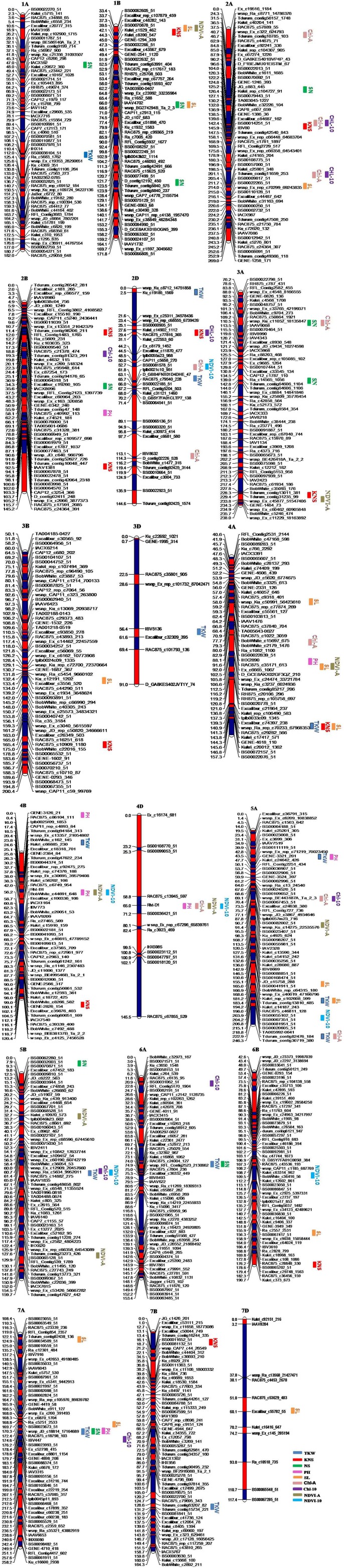
**Genetic maps of chromosomes showing QTL for yield components, plant height, and yield-related physiological traits in the Zhou 8425B/Chinese Spring population**. Traits are projected as solid bars with different colors for which the legend is given at the end of figure. TKW, thousand kernel weight; PH, plant height; SL, spike length; KNS, kernel number per spike; SN, spike number/m^2^; Chl-A, SPAD value of chlorophyll content at anthesis; Chl-10, SPAD value of chlorophyll content at 10 days post-anthesis; NDVI-A, normalized difference in vegetation index at anthesis; NDVI-10, normalized difference in vegetation index at 10 days post-anthesis.

ICIM identified 86 QTL for yield components, PH, and yield-related physiological traits based on data from individual location and year, and data averaged across the four environments. These QTL were detected on 18 chromosomes, excluding 1D, 5D, and 6D (Table 3, Figure 1). QTL for individual traits are described below.

**Table 3:** **QTL for yield components, plant height, and physiological traits in the Zhou8425B/Chinese Spring population**.

**Trait**	**Location and year**	**QTL**	**Position^a^**	**Marker interval**	**LOD^b^**	**PVE(%)^c^**	**Add^d^**
TKW	Zhoukou2013	*QTKW.caas-3DL*	57	*IBV5136—Excalibur_c32309_395*	3.16	3.9	−0.88
		*QTKW.caas-4AL*	141	*wsnp_Ra_rep_c70233_67968353—RAC875_c29282_566*	2.62	3.3	−0.8
		*QTKW.caas-4BS.1*	24	*BobWhite_c162_145—Kukri_c66885_230*	2.69	3.3	0.92
		*QTKW.caas-6A.1*	73.5	*Ku_c32392_967—wsnp_RFL_Contig2523_2130662*	5.98	7.3	−1.14
		*QTKW.caas-7AL*	172	*Kukri_rep_c97425_164—RAC875_c18798_103*	3.66	5.4	−1.05
	Zhengzhou2013	*QTKW.caas-1AL.4*	125	*BS00036104_51—Ra_c5683_1762*	4.33	5.1	−0.96
		*QTKW.caas-4AL*	141	*wsnp_Ra_rep_c70233_67968353—RAC875_c29282_566*	4.59	5.5	−1.27
		*QTKW.caas-5AL.1*	220	*TA005992-0641—Tdurum_contig82476_184*	3.35	4.5	1.37
		*QTKW.caas-6A.1*	73.5	*Ku_c32392_967—wsnp_RFL_Contig2523_2130662*	7.71	4.8	−1.03
		*QTKW.caas-7AL*	172	*Kukri_rep_c97425_164 —RAC875_c18798_103*	2.86	6.5	−1.19
		*QTKW.caas-7BL*	131	*Tdurum_contig63207_82—Tdurum_contig15734_221*	3.71	4.3	−0.86
	Zhoukou2014	*QTKW.caas-2DL.1*	65	*D_GBB4FNX01D4DHE_47—RAC875_c79540_228*	3.38	4.5	−0.82
		*QTKW.caas-5AS.1*	44.5	*wsnp_Ex_rep_c71219_70023450—Kukri_c24642_426*	2.81	3.4	−0.9
		*QTKW.caas-5BL*	61	*wsnp_Ra_c5634_9952011—RAC875_c14882_275*	6.54	8.2	−1.1
		*QTKW.caas-6A.1*	73.5	*Ku_c32392_967—wsnp_RFL_Contig2523_2130662*	7.71	9.9	−1.31
		*QTKW.caas-7AL*	172	*Kukri_rep_c97425_164—RAC875_c18798_103*	2.86	3.5	−0.81
		*QTKW.caas-7BL*	131	*Tdurum_contig63207_82—Tdurum_contig15734_221*	3.55	4.3	−1.05
	Zhengzhou2014	*QTKW.caas-2DL.2*	1	*wsnp_Ku_c8712_14751858—Ku_c19185_1569*	4.44	5.3	−1.03
		*QTKW.caas-4AL*	141	*wsnp_Ra_rep_c70233_67968353—RAC875_c29282_566*	2.96	3.4	−1.07
		*QTKW.caas-5AS.1*	44.5	*wsnp_Ex_rep_c71219_70023450—Kukri_c24642_426*	2.9	3.5	−0.98
		*QTKW.caas-5AL.2*	183	*Kukri_rep_c102608_599—Tdurum_contig13810_485*	5.12	6.0	−1.25
		*QTKW.caas-6A.1*	73.5	*Ku_c32392_967—wsnp_RFL_Contig2523_2130662*	7.82	9.2	−1.31
		*QTKW.caas-7AL*	172	*Kukri_rep_c97425_164—RAC875_c18798_103*	4.56	5.4	−1.09
		*QTKW.caas-7BL*	131	*Tdurum_contig63207_82—Tdurum_contig15734_221*	3.53	4.0	−1.05
	Average	*QTKW.caas-3DL*	57	*IBV5136—Excalibur_c32309_395*	5.37	5.8	−1.01
		*QTKW.caas-4AL*	141	*wsnp_Ra_rep_c70233_67968353—RAC875_c29282_566*	5.83	6.6	−0.97
		*QTKW.caas-5AL.2*	183	*Kukri_rep_c102608_599—Tdurum_contig13810_485*	3.95	4.2	−1.16
		*QTKW.caas-6A.1*	73.5	*Ku_c32392_967—wsnp_RFL_Contig2523_2130662*	9.3	10.3	−0.85
		*QTKW.caas-7AL*	172	*Kukri_rep_c97425_164—RAC875_c18798_103*	4.52	4.8	−1.01
		*QTKW.caas-7BL*	131	*Tdurum_contig63207_82—Tdurum_contig15734_221*	5.22	5.5	−0.96
KNS	Zhoukou2013	*QKNS.caas-2B.1*	92	*Tdurum_contig10048_447—IAAV1381*	2.9	5.7	−1.79
		*QKNS.caas-3AL*	228.3	*RAC875_c61934_186—wsnp_Ex_c45877_51547406*	6.98	10.9	2.46
		*QKNS.caas-4AL*	139	*Kukri_rep_c106490_583—RAC875_c29282_566*	3.39	5.1	−1.68
		*QKNS.caas-7BS*	18	*BS00011652_51—BS00081132_51*	3.02	4.6	1.6
	Zhengzhou2013	*QKNS.caas-3AL*	228.3	*RAC875_c61934_186—wsnp_Ex_c45877_51547406*	3.18	5.1	1.67
		*QKNS.caas-3B*	166	*RAC875_c10909_1180—BobWhite_c22016_155*	3.42	5.6	−1.75
		*QKNS.caas-4AL*	139	*Kukri_rep_c106490_583—RAC875_c29282_566*	6.44	10.5	−2.4
	Zhoukou2014	*QKNS.caas-2B.2*	12	*Tdurum_contig98206_211—RFL_Contig1483_1765*	3.45	4.4	−1.54
		*QKNS.caas-3AL*	228.3	*RAC875_c61934_186—wsnp_Ex_c45877_51547406*	2.79	3.4	1.35
		*QKNS.caas-3B*	166	*RAC875_c10909_1180—BobWhite_c22016_155*	3.32	4.1	−1.48
		*QKNS.caas-4AL*	139	*Kukri_rep_c106490_583—RAC875_c29282_566*	7.23	9.1	−2.22
		*QKNS.caas-4BL.1*	100	*BobWhite_c8266_582—GENE-2826_154*	3.7	4.7	−1.59
		*QKNS.caas-6BL*	178	*RAC875_c28848_330—BS00065202_51*	3.84	5.0	1.7
	Zhengzhou2014	*QKNS.caas-1BS.1*	43	*Kukri_c1529_462—Kukri_c8390_547*	4.13	7.5	1.83
		*QKNS.caas-2D*	29	*Kukri_c14902_1112—RAC875_c77816_365*	2.62	5.3	−1.29
		*QKNS.caas-4AL*	139	*Kukri_rep_c106490_583—RAC875_c29282_566*	6.42	9.4	−1.59
		*QKNS.caas-2AL.1*	143	*BS00014251_51—IBV80*	5.11	5.4	−1.29
	Average	*QKNS.caas-2B.1*	92	*Tdurum_contig10048_447—IAAV1381*	4.88	6.1	−1.37
		*QKNS.caas-3AL*	228.3	*RAC875_c61934_186—wsnp_Ex_c45877_51547406*	7	7.2	1.48
		*QKNS.caas-3B*	166	*RAC875_c10909_1180—BobWhite_c22016_155*	5.21	8.8	−1.83
		*QKNS.caas-4AL*	139	*Kukri_rep_c106490_583—RAC875_c29282_566*	10.93	11.8	−1.89
SN	Zhoukou2013	*QSN.caas-1AL.1*	47.5	*IACX592—Jagger_c1403_60*	7.66	13.8	−17.49
		*QSN.caas-3AL*	141	*Ra_c14565_1056—Tdurum_contig64606_1104*	2.63	6.4	−21.19
		*QSN.caas-7AL*	170	*BS00023673_51—wsnp_JD_c18814_17164689*	3.39	6.4	−20.53
	Zhengzhou2013	*QSN.caas-1AL.1*	47.5	*IACX592—Jagger_c1403_60*	8.59	11.4	−34.46
		*QSN.caas-1BL*	122	*D_contig12192_450—Tdurum_contig8840_575*	2.62	3.4	−18.77
		*QSN.caas-2BL*	35	*Excalibur_c19260_105—IACX8096*	3.63	6.7	26.32
		*QSN.caas-3AS*	113	*wsnp_Ku_c40218_48484410—wsnp_Ex_rep_c106152_90334299*	2.95	3.8	−19.75
	Zhoukou2014	*QSN.caas-1AL.1*	47.5	*IACX592—Jagger_c1403_60*	5.51	8.0	−20.08
		*QSN.caas-2AS.3*	126	*JG_c883_445—Kukri_rep_c104727_91*	3.91	5.6	16.75
		*QSN.caas-3AL*	141	*Ra_c14565_1056—Tdurum_contig64606_1104*	3.93	7.9	−20.07
		*QSN.caas-5BS.1*	9	*BS00032003_51—BS00070871_51*	2.92	4.3	14.69
		*QSN.caas-6AL.1*	75	*RAC875_c7804_236—Excalibur_c36332_449*	3.66	5.3	−16.19
	Zhengzhou2014	*QSN.caas-1AL.1*	47.5	*IACX592—Jagger_c1403_60*	15.09	17.2	−34.6
		*QSN.caas-1BL.1*	66	*IAAV4702—wsnp_BG274294B_Ta_2_3*	3.03	2.3	−12.98
		*QSN.caas-2AS.3*	126	*JG_c883_445—Kukri_rep_c104727_91*	3.81	3.0	14.31
		*QSN.caas-3AL*	141	*Ra_c14565_1056—Tdurum_contig64606_1104*	9	11.7	−29.26
		*QSN.caas-6AL.1*	75	*RAC875_c7804_236—Excalibur_c36332_449*	7.12	5.5	−19.5
		*QSN.caas-7AL*	170	*BS00023673_51—wsnp_JD_c18814_17164689*	6.89	5.9	−20.09
	Average	*QSN.caas-1AL.1*	47.5	*IACX592—Jagger_c1403_60*	12.09	14.2	−24.72
		*QSN.caas-2AS.3*	128	*Kukri_c7914_99—wsnp_Ex_c36242_44232305*	4.17	4.9	14.57
		*QSN.caas-3AL*	141	*Ra_c14565_1056—Tdurum_contig64606_1104*	6.93	11.1	−22.1
		*QSN.caas-6AL.1*	75	*RAC875_c7804_236—Excalibur_c36332_449*	6.33	5.8	−17.92
PH	Zhoukou2013	*QPH.caas-4AL*	90	*IHX2890—RAC875_c35171_613*	4.37	3.9	−2.56
		*QPH.caas-4BS.1*	11	*RAC875_c86104_111—tplb0025f09_1853*	4.88	4.9	−2.87
		*QPH.caas-4BS.2*	55	*RAC875_c6749_954—BobWhite_c44691_648*	21.68	22.8	−6.35
		*QPH.caas-4DS.1*	63.8	*RAC875_c13945_597—BS00036421_51*	13.66	14.5	−4.99
		*QPH.caas-5AS*	50	*Kukri_c24642_426—RFL_Contig2251_434*	5.67	14.9	−2.89
		*QPH.caas-7AL*	170	*BS00023673_51—wsnp_JD_c18814_17164689*	2.99	2.6	2.1
	Zhengzhou2013	*QPH.caas-4BS.2*	55	*RAC875_c6749_954—BobWhite_c44691_648*	14.87	22.7	−6.67
		*QPH.caas-4DS.1*	63.8	*RAC875_c13945_597—BS00036421_51*	14.02	23.5	−6.71
		*QPH.caas-5AS*	50	*Kukri_c24642_426—RFL_Contig2251_434*	5.84	13.2	−2.61
	Zhoukou2014	*QPH.caas-2BL*	58	*Tdurum_contig47_148—RAC875_c40992_113*	2.62	2.3	2.4
		*QPH.caas-4AL*	90	*IHX2890—RAC875_c35171_613*	3.25	2.9	−2.69
		*QPH.caas-4BS.2*	55	*RAC875_c6749_954—BobWhite_c44691_648*	22.59	24.2	−7.98
		*QPH.caas-4DS.1*	63.8	*RAC875_c13945_597—BS00036421_51*	26.88	33.2	−9.2
		*QPH.caas-5AS*	50	*Kukri_c24642_426—RFL_Contig2251_434*	5.67	14.3	−2.71
	Zhengzhou2014	*QPH.caas-2BL*	58	*Tdurum_contig47_148—RAC875_c40992_113*	2.82	2.8	2.93
		*QPH.caas-4BS.2*	55	*RAC875_c6749_954—BobWhite_c44691_648*	23.1	29.3	−9.58
		*QPH.caas-4DS.1*	63.8	*RAC875_c13945_597—BS00036421_51*	21.21	29.6	−9.51
	Average	*QPH.caas-4AL*	90	*IHX2890—RAC875_c35171_613*	2.73	2.6	−2.26
		*QPH.caas-4BS.2*	55	*RAC875_c6749_954—BobWhite_c44691_648*	24.73	27.4	−7.53
		*QPH.caas-4DS.1*	63.8	*RAC875_c13945_597—BS00036421_51*	24.01	30.0	−7.78
		*QPH.caas-5AS*	50	*Kukri_c24642_426—RFL_Contig2251_434*	2.95	12.7	−2.31
SL	Zhoukou2013	*QSL.caas-1BL*	66	*IAAV4702—wsnp_BG274294B_Ta_2_3*	4.86	6.0	0.34
		*QSL.caas-4AS*	57.6	*Kukri_c46057_646—RAC875_rep_c77874_269*	3.68	4.5	0.28
		*QSL.caas-4AL.1*	146	*Kukri_c17417_571—BS00022076_51*	7.93	11.2	−0.44
		*QSL.caas-5AL*	157.5	*JD_c15758_288—BS00041911_51*	5.78	8.7	−0.39
		*QSL.caas-7AS*	168	*wsnp_Ex_c200_391493—Ex_c6870_1704*	4.1	4.9	0.29
		*QSL.caas-7DS*	60	*RAC875_c53629_483—Excalibur_c55782_55*	3.27	4.0	0.27
	Zhengzhou2013	*QSL.caas-1BL*	66	*IAAV4702—wsnp_BG274294B_Ta_2_3*	2.6	3.6	0.24
		*QSL.caas-3BL*	133	*Ku_c12191_1202—Excalibur_c3556_520*	2.77	3.9	0.24
		*QSL.caas-4AS*	57.6	*Kukri_c46057_646—RAC875_rep_c77874_269*	7.98	12.3	0.43
		*QSL.caas-4AL.1*	146	*Kukri_c17417_571—BS00022076_51*	4.67	6.8	−0.32
		*QSL.caas-5AL*	157.5	*JD_c15758_288—BS00041911_51*	4.24	6.4	−0.31
	Zhoukou2014	*QSL.caas-2AL*	212.5	*BS00022265_51—wsnp_Ex_rep_c70299_69243835*	3.54	4.3	0.26
		*QSL.caas-4AS*	57.6	*Kukri_c46057_646—RAC875_rep_c77874_269*	8.53	10.3	0.41
		*QSL.caas-4AL.1*	146	*Kukri_c17417_571—BS00022076_51*	9.52	11.8	−0.43
		*QSL.caas-5AL.1*	183.5	*Kukri_rep_c102608_599—Kukri_c14187_243*	7.09	8.7	−0.38
		*QSL.caas-6BL*	156	*Ra_c2557_2531—BS00067417_51*	3.16	3.6	0.26
		*QSL.caas-7AS.1*	124	*Tdurum_contig82438_136—BS00034509_51*	4.26	9.6	0.45
	Zhengzhou2014	*QSL.caas-1BS*	42	*BS00070878_51—Kukri_c1529_462*	4.79	5.2	0.36
		*QSL.caas-2AL*	212.5	*BS00022265_51–wsnp_Ex_rep_c70299_69243835*	3.65	4.0	0.28
		*QSL.caas-4AS*	57.6	*Kukri_c46057_646–RAC875_rep_c77874_269*	7.58	8.6	0.4
		*QSL.caas-4AL.2*	98	*Ex_c6665_1067—D_GCE8AKX02GF3QZ_210*	3.03	5.4	0.32
		*QSL.caas-4AL.1*	146	*Kukri_c17417_571—BS00022076_51*	9.81	11.9	−0.48
		*QSL.caas-5AL.1*	183.5	*Kukri_rep_c102608_599—Kukri_c14187_243*	5.37	6.0	−0.34
		*QSL.caas-6BL*	156	*Ra_c2557_2531—BS00067417_51*	2.91	3.1	0.26
		*QSL.caas-7AS*	168	*wsnp_Ex_c200_391493—Ex_c6870_1704*	3.58	5.4	0.33
	Average	*QSL.caas-1BS*	43	*Kukri_c1529_462–Kukri_c8390_547*	6.3	8.8	0.44
		*QSL.caas-4AS*	57.6	*Kukri_c46057_646—RAC875_rep_c77874_269*	11.24	13.3	0.42
		*QSL.caas-4AL.1*	146	*Kukri_c17417_571—BS00022076_51*	11.91	14.9	−0.44
		*QSL.caas-4AL.2*	98	*Ex_c6665_1067—D_GCE8AKX02GF3QZ_210*	2.61	2.7	0.19
		*QSL.caas-5AL*	159	*JD_c15758_288—BS00041911_51*	6.89	9.1	−0.35
		*QSL.caas-7AS*	168	*wsnp_Ex_c200_391493—Ex_c6870_1704*	4.55	4.8	0.25
Chl-A	Zhoukou2013	*QChl-A.caas-2AS*	45	*wsnp_Ex_c322_624793—Tdurum_contig10785_103*	2.59	4.5	0.51
		*QChl-A.caas-3AS*	111	*wsnp_Ku_c11052_18135847— wsnp_Ra_c16278_24893033*	3.46	5.6	0.56
		*QChl-A.caas-5AL*	68.5	*BS00109052_51—wsnp_BE443187A_Ta_2_3*	3.89	6.5	0.6
	Zhengzhou2013	*QChl-A.caas-2AL.1*	199	*Kukri_c25901_348—Tdurum_contig11659_253*	3.42	5.1	0.71
		*QChl-A.caas-2DS*	55	*BS00081578_51—tplb0021c10_951*	3.75	8.6	0.92
		*QChl-A.caas-3AS*	111	*wsnp_Ku_c11052_18135847—wsnp_Ra_c16278_24893033*	4.31	6.5	0.8
		*QChl-A.caas-5AL*	68.5	*BS00109052_51—wsnp_BE443187A_Ta_2_3*	4.33	16.3	0.72
	Zhoukou2014	*QChl-A.caas-4AL*	78	*BobWhite_c15697_675—BobWhite_c2179_1476*	3.56	7.1	0.92
		*QChl-A.caas-4DS*	67	*RAC875_c13945_597—BS00036421_51*	3.49	6.3	0.86
		*QChl-A.caas-5AL*	68.5	*BS00109052_51—wsnp_BE443187A_Ta_2_3*	5.55	8.8	1.01
	Zhengzhou2014	*QChl-A.caas-2AL.2*	140	*Excalibur_c84687_162—BS00014251_51*	3.94	5.7	−0.82
		*QChl-A.caas-2DL.1*	114	*IBV8632—D_contig02226_528*	2.75	3.9	0.68
		*QChl-A.caas-5AL*	68.5	*BS00109052_51—wsnp_BE443187A_Ta_2_3*	5.36	7.8	0.96
		*QChl-A.caas-5AS.1*	227	*Tdurum_contig82476_184—Tdurum_contig30719_380*	3.01	8.4	1.31
	Average	*QChl-A.caas-2AL.2*	140	*Excalibur_c84687_162— BS00014251_51*	4.27	6.0	−0.61
		*QChl-A.caas-2AL.1*	199	*Kukri_c25901_348— Tdurum_contig11659_253*	3.53	4.8	0.54
		*QChl-A.caas-3AS*	111	*wsnp_Ku_c11052_18135847—wsnp_Ra_c16278_24893033*	3.58	4.9	0.55
		*QChl-A.caas-4DS*	64	*RAC875_c13945_597—BS00036421_51*	4.04	6.2	0.63
		*QChl-A.caas-5AL*	68.5	*BS00109052_51—wsnp_BE443187A_Ta_2_3*	6.6	9.2	0.75
Chl-10	Zhoukou2013	*QChl-10.caas-2D*	29	*Kukri_c14902_1112—RAC875_c77816_365*	4.11	5.5	−1.41
		*QChl-10.caas-5BL*	60	*wsnp_Ex_c12909_20457660—wsnp_Ra_c5634_9952011*	4.83	8.2	1.78
	Zhengzhou2013	*QChl-10.caas-2BS*	19	*RAC875_c21378_474—Tdurum_contig81323_291*	3.78	6.3	−1.25
		*QChl-10.caas-5BL*	60	*wsnp_Ex_c12909_20457660—wsnp_Ra_c5634_9952011*	6.06	10.3	1.59
	Zhoukou2014	*QChl-10.caas-2D*	29	*Kukri_c14902_1112—RAC875_c77816_365*	4.04	9.2	−1.6
		*QChl-10.caas-5BL*	60	*wsnp_Ex_c12909_20457660— wsnp_Ra_c5634_9952011*	4.83	7.0	1.33
		*QChl-10.caas-6AS*	21	*BS00031062_51—RFL_Contig5170_1904*	3.07	4.3	−1
		*QChl-10.caas-7A*	177	*BS00023993_51—Ex_c52798_415*	3.98	7.9	1.38
	Zhengzhou2014	*QChl-10.caas-2AL*	140	*Excalibur_c84687_162—BS00014251_51*	5.44	7.0	−0.91
		*QChl-10.caas-2AL.1*	200	*Kukri_c25901_348—Tdurum_contig11659_253*	4.77	6.1	0.85
		*QChl-10.caas-2BS*	19	*RAC875_c21378_474—Tdurum_contig81323_291*	5.37	7.8	−0.96
		*QChl-10.caas-5AL*	72	*BS00067453_51—Excalibur_c24638_380*	4.89	6.8	0.9
		*QChl-10.caas-5BL*	60	*wsnp_Ex_c12909_20457660—wsnp_Ra_c5634_9952011*	8.09	10.6	1.13
		*QChl-10.caas-7A*	177	*BS00023993_51—Ex_c52798_415*	2.53	4.2	0.72
	Average	*QChl-10.caas-2BS*	19	*RAC875_c21378_474—Tdurum_contig81323_291*	5.31	8.3	−1.14
		*QChl-10.caas-5AL*	77	*RFL_Contig727_736—wsnp_JD_c3867_4934646*	2.71	3.5	0.73
		*QChl-10.caas-5BL*	60	*wsnp_Ex_c12909_20457660—wsnp_Ra_c5634_9952011*	10.19	14.2	1.49
		*QChl-10.caas-7A*	177	*BS00023993_51—Ex_c52798_415*	3.14	5.7	0.96
NDVI-A	Zhengzhou2013	*QNDVI-A.caas-4AL*	93	*Ex_c6665_1067—D_GCE8AKX02GF3QZ_210*	3.27	6.5	0.01
		*QNDVI-A.caas-5BL*	128	*Tdurum_contig23273_426—BS00065128_51*	3.74	6.5	−0.01
	Zhoukou2014	*QNDVI-A.caas-3AL*	230	*Tdurum_contig31235_99—wsnp_Ex_c45877_51547406*	2.9	4.7	0.01
		*QNDVI-A.caas-5BS.1*	33	*Kukri_c10970_573—tplb0046h23_602*	3.46	5.7	0.01
	Zhengzhou2014	*QNDVI-A.caas-1BS*	41	*BS00070878_51—Kukri_c1529_462*	3.88	6.7	0.01
		*QNDVI-A.caas-4BS*	56	*RAC875_c6749_954—BobWhite_c44691_648*	2.73	4.0	0.01
		*QNDVI-A.caas-4DS*	69	*RAC875_c13945_597—BS00036421_51*	6.03	9.8	0.01
		*QNDVI-A.caas-5AL*	91	*BS00082002_51—wsnp_Ku_c14275_22535576*	4.15	6.4	0.01
	Average	*QNDVI-A.caas-3AL*	230	*Tdurum_contig31235_99—wsnp_Ex_c45877_51547406*	2.54	4.3	0.01
NDVI-10	Zhoukou2013	*QNDVI-10.caas-2DS*	53	*BS00081578_51—tplb0021c10_951*	2.67	4.4	−0.02
		*QNDVI-10.caas-5AL*	204	*BS00055102_51—BS00067351_51*	3.94	6.3	−0.02
		*QNDVI-10.caas-5BL*	61	*wsnp_Ra_c5634_9952011—RAC875_c14882_275*	5.2	8.5	0.02
	Zhengzhou2013	*QNDVI-10.caas-4BS*	53	*RAC875_c6749_954— BobWhite_c44691_648*	2.93	5.5	0.02
		*QNDVI-10.caas-5BL*	61	*wsnp_Ra_c5634_9952011—RAC875_c14882_275*	3.52	6.1	0.02
	Zhoukou2014	*QNDVI-10.caas-5BL*	61	*wsnp_Ra_c5634_9952011—RAC875_c14882_275*	3.69	6.0	0.01
		*QNDVI-10.caas-6BL*	109	*Kukri_c63314_962—BobWhite_c36416_56*	3.37	5.8	0.01
	Zhengzhou2014	*QNDVI-10.caas-4DS*	68	*RAC875_c13945_597—BS00036421_51*	3.48	7.0	0.01
		*QNDVI-10.caas-6BL*	109	*Kukri_c63314_962—BobWhite_c36416_56*	3.86	7.3	0.01
	Averages	*QNDVI-10.caas-5BL*	61	*wsnp_Ra_c5634_9952011—RAC875_c14882_275*	5.09	8.5	0.02
		*QNDVI-10.caas-6BL*	109	*Kukri_c63314_962—BobWhite_c36416_56*	3.34	6.3	0.01

a *Position of QTL located on chromosome: as cM distance from the top of each map*.

b *A LOD threshold of 2.5 was used for declaration of QTL, based on 2000 permutations at a significance level of 0.01*.

c *Phenotypic variance explained by QTL*.

d *Positive “additive effect” indicates an increasing effect from Chinese Spring; negative “additive effect” indicates an increasing effect from Zhou 8425B*.

*TKW, thousand kernel weight; KNS, kernel number per spike; SN, spike number/m^2^; PH, plant height; SL, spike length; Chl-A, SPAD value of chlorophyll content at anthesis; Chl-10, SPAD value of chlorophyll content at 10 days post-anthesis; NDVI-A, normalized difference in vegetation index at anthesis; NDVI-10, normalized difference in vegetation index at 10 days post-anthesis*.

### Thousand kernel weight

Thirteen QTL for TKW were identified on chromosomes 1AL, 2DL (2), 3DL, 4AL, 4BS, 5AL (2), 5AS, 5BL, 6A, 7AL, and 7BL, respectively (Table 3, Figure 1). Alleles that increased TKW at 11 loci were derived from the parent Zhou 8425B, however two positive alleles were contributed by Chinese Spring. *QTKW.caas-6A.1* and *QTKW.caas-7AL* were stably identified across all environments, and explained 4.8–10.3% and 3.5–6.5% of the phenotypic variances, respectively. Two other QTL, *QTKW.caas-4AL*, and *QTKW.caas-7BL*, were detected in three environments, explaining 3.3–6.6% and 4.0–5.5% of the phenotypic variances, respectively. *QTKW.caas-5AS.1* was detected at Zhoukou2014 and Zhengzhou2014, and explained from 3.4 to 3.5% of the phenotypic variance. The positive alleles at *QTKW.caas-1AL.4, QTKW.caas-2DL.1, QTKW.caas-2DL.2, QTKW.caas-3DL, QTKW.caas-4AL, QTKW.caas-5AL.2, QTKW.caas-5AS.1, QTKW.caas-5BL, QTKW.caas-6A.1, QTKW.caas-7AL*, and *QTKW.caas-7BL* loci were contributed by Zhou 8425B. Those for increasing TKW at *QTKW.caas-4BS.1* and *QTKW.caas-5AL.1* loci were derived from Chinese Spring.

### Kernel number per spike

Eleven QTL for KNS were identified on chromosomes 1BS, 2AL, 2B (2), 2D, 3AL, 3B, 4AL, 4BL, 6BL, and 7BS (Table 3, Figure 1). Alleles increasing KNS at the loci on chromosomes 2AL, 2B (2), 2D, 3B, 4AL, and 4BL were contributed by Zhou 8425B, and those on chromosomes 1BS, 3AL, 6BL, and 7BS were come from Chinese Spring. *QKNS.caas-4AL* flanked by SNP markers *Kukri_rep_c106490_583* and *RAC875_c29282_566* was detected in all environments, explaining 5.1–10.5% of the phenotypic variance. The second QTL between SNP markers *RAC875_c61934_186* and *wsnp_Ex_c45877_51547406* on chromosome 3AL was found in three environments, and explained 3.4–10.9% of the phenotypic variance. *QKNS.caas-3B* detected at Zhengzhou2013 and Zhoukou2014 accounted for 4.1–5.6% of the phenotypic variance.

### Spike number

Ten QTL for SN were identified on chromosomes 1AL, 1BL (2), 2AS, 2BL, 3AL, 3AS, 5BS, 6AL, and 7AL (Table 3, Figure 1). Alleles for increased SN present on chromosomes 1AL, 1BL, 3AL, 3AS, 6AL, and 7AL were contributed by Zhou 8425B, and positive alleles on 2AS, 2BL and 5BS came from Chinese Spring. *QSN.caas-1AL.1* between the SNP markers *IACX592* and *Jagger_c1403_60* was identified in all four environments, explained 8.0–17.2% of the variation in SN. Another QTL, *QSN.caas-3AL*, between markers *Ra_c14565_1056* and *Tdurum_contig64606_1104*, was identified in three environments and explained 6.4–11.7% of the phenotypic variance. *QSN.caas-2AS.3, QSN.caas-6AL.1* and *QSN.caas-7AL* were detected in two environments and explained 3.0–6.4% of the phenotypic variance.

### Plant height

Seven QTL for PH were detected on chromosomes 2BL, 4AL, 4BS (2), 4DS, 5AS, and 7AL, respectively (Table 3, Figure 1). The alleles reducing PH on 4AL, 4BS, 4DS, and 5AS came from the shorter parent Zhou 8425B and those on 2BL and 7AL were from Chinese Spring. *QPH.caas-4BS.2* flanked by markers *RAC875_c6749_954* and *BobWhite_c44691_648* was identified in all four environments, and explained 22.7–29.3% of the phenotypic variance. *QPH.caas-4DS.1* between markers *RAC875_c13945_597* and *BS00036421_51* was also detected across all environments, accounting for 14.5–33.2% of the PH variance. These two major QTL are *Rht-B1b* and *Rht-D1b*, respectively, based on the gene-specific markers. *QPH.caas-5AS* was found in three environments and explained 12.7–14.9% of the phenotypic variance. *QPH.caas-2BL* and *QPH.caas-4AL* were consistently observed in two environments and explained 2.3–3.9% of the phenotypic variance.

### Spike length

Thirteen QTL for SL were mapped on chromosomes 1BL, 1BS, 2AL, 3BL, 4AS, 4AL (2), 5AL (2), 6BL, 7AS (2), and 7DS (Table 3, Figure 1). Alleles for increased SL at the loci on chromosomes 4AL (1) and 5AL were contributed by Zhou 8425B, and those at loci on chromosomes 1B, 2AL, 3BL, 4AS, 4AL (1), 6BL, 7AS (2), and 7DS were from Chinese Spring. *QSL.caas-4AS* between markers *Kukri_c46057_646* and *RAC875_rep_c77874_269* was detected in all environments, explaining 4.5–12.3% of the phenotypic variance. *QSL.caas-4AL.1* flanked by SNP markers *Kukri_c17417_571* and *BS00022076_51* was also found in four environments, accounting for 6.8–11.9% of the phenotypic variance. *QSL.caas-1BL, QSL.caas-2AL, QSL.caas-5AL, QSL.caas-5AL.1, QSL.caas-6BL*, and *QSL.caas-7AS* detected in two environments explained 3.1–8.7% of the phenotypic variance.

### SPAD value of chlorophyll content at anthesis

Ten QTL for Chl-A were detected on chromosomes 2AS, 2AL (2), 2DS, 2DL, 3AS, 4AL, 4DS, 5AS, and 5AL (Table 3, Figure 1). Alleles increasing Chl-A at all loci except *QChl-A.caas-2AL.2* were contributed by Chinese Spring. *QChl-A.caas-5AL* flanked by SNP markers *BS00109052_51* and *wsnp_BE443187A_Ta_2_3* was detected in all environments, explaining 6.5–16.3% of the phenotypic variance. *QChl-A.caas-3AS* between markers *wsnp_Ku_c11052_18135847* and *wsnp_Ra_c16278_24893033* was identified in two environments**, explaining 5.6–6.5% of the phenotypic variance.

### SPAD value of chlorophyll content at 10 days post-anthesis

Eight putative QTL for Chl-10 were detected on chromosomes 2AL (2), 2BS, 2D, 5AL, 5BL, 6AS, and 7A (Table 3, Figure 1). *QChl-10.caas-5BL* between SNP markers *wsnp_Ex_c12909_20457660* and *wsnp_Ra_c5634_9952011* was detected in all four environments, explaining 7.0–10.6% of the phenotypic variance; the additive effect was for the Chinese Spring allele. *QChl-10.caas-2BS, QChl-10.caas-2D* and *QChl-10.caas-7A* were significant in two environments and explained 4.2–9.2% of the phenotypic variance. Alleles increasing Chl-10 at the *QChl-10.caas-2BS* and *QChl-10.caas-2D* loci came from Zhou 8425B, whereas that increasing Chl-10 at *QChl-10.caas-7A* locus was derived from Chinese Spring.

### Normalized difference in vegetation index at anthesis

Eight QTL for NDVI-A were detected on chromosomes 1BS, 3AL, 4AL, 4BS, 4DS, 5AL, 5BL, and 5BS (Table 3, Figure 1), explaining 4.0–9.8% of the phenotypic variances. The alleles increasing NDVI-A at all loci except *QNDVI-A.caas-5BL* were from Chinese Spring.

### Normalized difference in vegetation index at 10 days post-anthesis

Six QTL for NDVI-10 were identified on chromosomes 2DS, 4BS, 4DS, 5AL, 5BL, and 6BL (Table 3, Figure 1). Alleles for increasing NDVI-10 at the loci on chromosomes 2DS and 5AL were contributed by Zhou 8425B, and those at other loci were from Chinese Spring. *QNDVI-10.caas-5BL* flanked by markers *wsnp_Ra_c5634_9952011* and *RAC875_c14882_275* was significant in three environments and explained 6.0–8.5% of the phenotypic variance; the positive allele came from Chinese Spring. *QNDVI-10.caas-6BL* was found in two environments and explained 5.8–7.3% of the phenotypic variance.

## Discussion

### SNP discovery and linkage map construction

SNP markers enable construction of high-density linkage maps and identification of QTL for complex agronomic traits in crop plants (Song et al., 2013). In the current study, we used 5636 polymorphic SNP markers from a 90K SNP assay (Wang et al., 2014), and constructed a high-density genetic map for a RIL population derived from the cross Zhou 8425B/Chinese Spring. Of these markers, 4770 (84.6%) were mapped by Wang et al. (2014) and 866 are newly mapped (Table S4). The order of SNP markers in the linkage map is generally consistent with Wang et al. (2014). The total length of the linkage map was 3609.4 cM, similar to previously reported maps in hexaploid wheat (Blanco et al., 1998; Marone et al., 2012). The average density of the map was 0.64 cM/marker, representing a considerable improvement over previously reported maps based on SSR, STS and DArT (Nachit et al., 2001; Marone et al., 2012). Markers for the A (43.6%) and B (50.4%) genomes were more abundant than those for the D genome (6%), again consistent with previous studies (Shiaoman et al., 2009; Wang et al., 2014), and this is attributed to the low level of polymorphism in D genome of hexaploid wheat. Although the average density is high, there were still some gaps, for instance, on chromosomes 1B and 4D.

The average number of mapped markers per chromosome was 268.4, ranging from 10 on chromosome 3D to 599 on chromosome 5B. However, 65.6% of the SNPs mapped displayed redundancy and only 6.9% of SNPs were used for linkage map construction in the present study, in agreement with previous reports (Barker and Edwards, 2009; Colasuonno et al., 2014). The low polymorphisms of SNPs in high-density assays identified in these studies may reflect an overall narrow range of genetic diversity in wheat. Because many SNP markers were co-located at the same genetic loci (Colasuonno et al., 2014), the BIN-Mapping function was employed in selecting markers for QTL mapping. BIN helps with automatic deletion of the high “nearest neighbor” markers in generating the input file that can be used for more efficient genetic map construction. The 5636 polymorphic SNP markers were optimized by the BIN-Mapping function and a linkage map based on 1938 skeleton SNPs were used to perform QTL analysis. Indeed, this generated a simplified genetic map for QTL mapping.

### QTL mapping

The green-revolution genes, *Rht1* and *Rht2* on chromosomes 4B and 4D, respectively, have been deployed worldwide (Ellis et al., 2005; Peng et al., 2011). In the present study, the most interesting QTL associated with PH were also detected on chromosomes 4B and 4D across all environments. *QPH.caas-4BS.2* and *QPH.caas-4DS.1* carrying positive alleles from the short parent Zhou 8425B explained the highest phenotypic variance, and represent polymorphisms was associated with *Rht-B1* and *Rht-D1*. Another stable QTL for PH, *QPH.caas-5AS*, was positioned at 50 cM. A QTL for PH reported previously on chromosome 5A in a spring wheat population derived from a Seri/Babax cross based on SSR marker *Xgwm617a* (Lopes et al., 2013) is likely the same gene. *QPH.caas-5AS* was detected in four environments and the reducing height allele was from Zhou 8425B. The gene could be used in MAS in wheat breeding. A minor PH QTL, *QPH.caas-4AL*, positioned at 90 cM, is different from a major QTL for PH at position 13.6cM on chromosome 4A in the Seri/Babax RIL population (Lopes et al., 2013). *QPH.caas-4AL*was detected in two environments and it is likely to be a new PH QTL.

An important QTL for TKW, *QTKW.caas-6A.1*, tightly linked to the SNP marker Ku_c32392_967 at a genetic distance of 1.4 cM was mapped at a similar locus to a QTL reported by Sukumaran et al. (2015) based on wheat 90K_consensus_map (Wang et al., 2014). *QTKW.caas-7BL*, positioned at 131cM across all four environments, is likely to be new. A minor QTL for TKW reported by Liu et al. (2014) in marker interval *Xcau30-7B-Xgwm66c-7B* positioned at 2cM in a mapping population derived from a cross of common wheat line ND3331 and Tibetan semi-wild wheat accession Zang1817 and is clearly different.

*QKNS.caas-4AL* mapped at 141 cM on chromosome 4AL in the present study, whereas Lopes et al. (2013) detected a different QTL for KNS at 5.55 cM on chromosome 4AS across 12 environments based on linkage with marker *C14p6*. A previously reported minor QTL for KNS (Liu et al., 2014) on chromosome 4AS, positioned at 0 cM, was also found in two environments but with very low contributions to phenotypic variation. Therefore, *QKNS.caas-4AL*, detected across all environments with higher phenotypic variation explained, is probably a new QTL. A new QTL *QKNS.caas-3AL* positioned at 228.3 cM in the current study differed from a major QTL at 56 cM reported by Ali et al. (2011) using a Cheyenne (CNN) × [CNN (Wichita 3A)] recombinant inbred chromosome line (RICL) population consisting of 223 CNN (RICLs3A) and seven check cultivars.

*QSN.caas-1AL.1*, at 47.5cM on chromosome 1AL across all environments explained more than 10% of the phenotypic variance, was different from a minor QTL for SN reported in a Chuang 35,050/Shannong 483 RIL population using SSR markers (Li et al., 2007b); the latter was found in single environment with lower explained phenotypic variation (5.7%) and it is likely to be a new QTL. Another *QSN.caas-3AL* at about 141 cM and detected in three environments is different from a minor QTL for SN on chromosome 3AL based on the linked markers *Xgwm-720* and *Xgwm-1063* positioned at 44.5cM (Kumar et al., 2007). Therefore, *QSN.caas-3AL* is also new.

Only a few QTL for physiological traits have been detected in wheat (Rebetzke et al., 2008a,b; Reynolds and Tuberosam, 2008). A QTL for Chl-10 was mapped 60 cM on chromosome 5BL in this study, whereas a major QTL for Chl was previously identified between *Xgwm639.1-5B* and *Xwmc388.4-5B* (Xu et al., 2012). A minor QTL, *QChl-10.caas-6AS*, tightly linked to the SNP marker *RFL_Contig5170_1904* at a genetic distance of 1.8 cM was different from that reported by Sukumaran et al. (2015) due to the large genetic distance between the two linked SNP markers (Wang et al., 2014). *QChl-10.caas-5AL* located on the long arm of chromosome 5A across all environments has not been reported previously, therefore, it is a new QTL.

A significant QTL, QNDVI-10.caas-*5BL*, was tightly linked to the SNP marker *RAC875_c14882_275*, at a genetic distance of 0.4 cM, which may be different from that reported by Sukumaran et al. (2015) based on wheat 90K_consensus_map (Wang et al., 2014).

### Co-localization of QTL for yield and related traits

Co-localization of QTL or QTL clusters for yield and related traits have been reported in previous studies (McCartney et al., 2005; Quarrie et al., 2006). In the current study, 10 QTL clusters on chromosomes 1BS, 2AL (2), 3AL, 4AL (2), 4BS, 4DS, 5BL, and 7AL were detected with each for more than two traits (Table 4, Figure 1) and QTL clusters associated with GY were detected on chromosomes 3AL, 4DS, and 5BL.

**Table 4:** **Summary of pleiotropic QTL detected in the Zhou 8425B/Chinese Spring population**.

**Chromosome**	**Marker interval**	**Position (cM)**	**Trait**
1BS	*BS00070878_51~Kukri_c8390_547*	39.5~44.3	KNS, SL, NDVI-A
2AL	*Excalibur_c84687_162~IBV80*	139.6~145.9	KNS, Chl-A, Chl-10
2AL	*Kukri_c25901_348~wsnp_Ex_rep_c70299_69243835*	198.5~214.0	SL, Chl-A, Chl-10
3AL	*RAC875_c61934_186~wsnp_Ex_c45877_51547406*	217.4~233.8	GY, KNS, NDVI-A
4AL	*IHX2890~D_GCE8AKX02GF3QZ_210*	88.1~102.5	PH, SL, NDVI-A
4AL	*Kukri_rep_c106490_583~tplb0033c09_1345*	136.8~157.3	TKW, KNS, SL
4BS	*RAC875_c6749_954~BobWhite_c44691_648*	42.0~56.2	PH, NDVI-A, NDVI-10
4DS	*RAC875_c13945_597~BS00036421_51*	58.8~71.2	GY, PH, Chl-A, NDVI-A, NDVI-10
5BL	*wsnp_Ex_c10842_17637744~RAC875_c14882_275*	54.1~61.4	GY, TKW, Chl-10, NDVI-10
7AL	*wsnp_Ex_c200_391493~Ex_c52798_415*	168.0~178.4	TKW, SN, PH, SL, Chl-10

*GY, grain yield; TKW, thousand kernel weight; PH, plant height; SL, spike length; KNS, kernel number per spike; SN, spike number/m^2^; Chl-A, SPAD value of chlorophyll content at anthesis; Chl-10, SPAD value of chlorophyll content at 10 days post-anthesis; NDVI-A, normalized difference in vegetation index at anthesis; NDVI-10, normalized difference in vegetation index at 10 days post-anthesis*.

The interval 217.4–233.8 cM on chromosome 3AL is a pleiotropic locus impacting GY, KNS and NDVI-A. No similar pleiotropic region on chromosome 3A was reported previously. GY showed a significantly positive correlation with KNS (*r* = 0.44) and NDVI-A (*r* = 0.6), indicating that the increased GY at *QGY.caas-3AL* resulted from increased KNS and NDVI-A.

Another pleiotropic locus for GY, PH, Chl-A, NDVI-A, and NDVI-10 was identified at position 65 cM on chromosome 4DS. Co-localized QTL for GY and PH on chromosome 4DS detected in the present study and also reported by Li et al. (2015) are was associated with *Rht-D1b*. However, the co-localization of QTL for GY and Chl-A, NDVI-A, and NDVI-10 in same or similar region on chromosome 4DS has not been reported before.

QTL for GY, TKW, Chl-10, and NDVI-10 were associated in an interval of 54.1–61.4 cM on chromosome 5BL. A similar previously reported multiple trait region for GY and TKW on 5B (Edae et al., 2014) was positioned at 67.7–76.4 cM based on DArT markers in CIMMYT spring wheat lines. However, yield-related QTL for Chl-10 and NDVI-10 in a similar region on chromosome 5BL were not reported previously. Physiological traits Chl and NDVI were significantly correlated with GY in this study, with correlation coefficients of 0.5 and 0.34, respectively, implying that they may be related to the transfer of photosynthetic products in the grain filling (Lupton, 1966).

A QTL cluster for TKW, KNS and SL between markers *Kukri_rep_c106490_583* and *tplb0033c09_1345* and positioned in the interval 136.8–157.3 cM on chromosome 4AL, is likely the same or similar to a QTL cluster for KNS and SL reported by Liu et al. (2014) based on the interval *Xwmc491–Xwmc96*. Another QTL-rich region for PH, SL and NDVI-A, in interval 88.1–102.5 cM on chromosome 4AL, is new. *QTKW.caas-7AL* associated with SN, PH, SL, Chl-10, in interval 136.8–157.3 cM, was not reported previously.

In this study, a QTL-rich region for PH, NDVI-A and NDVI-10 on chromosome 4BS identified in interval 42.0–56.2 cM was different from the QTL for TKW and PH reported previously (Huang et al., 2004; McCartney et al., 2005). The 4BS QTL had a strong effect on PH and this QTL-rich region was associated with *Rht-B1b*.

### Potential application of QTL for MAS in wheat breeding

GY is highly affected by environments, and it is difficult to select high-yielding lines in smaller plots at the early stage of a breeding program. In contrast, environments have much less influence on yield components, PH and physiological traits, and some more stable QTL for these traits have been found, in agreement with previous reports (Lopes et al., 2013; Edae et al., 2014; Liu et al., 2014). Furthermore, yield was significantly and positively correlated with TKW, KNS, Chl-A, Chl-10, NDVI-A, and NDVI-10. Consequently, it is feasible to improve GY by selecting these yield-related traits in breeding programs because of the more accurate measurement and repeatability across environments in comparison with yield. Stable QTL such as *QTKW.caas-6A.1, QTKW.caas-7AL, QPH.caas-4BS.2, QPH.caas-4DS.1, QKNS.caas-3AL, QKNS.caas-4AL, QChl-A.caas-5AL, QChl-10.caas-5BL*, and *QNDVI-10.caas-5BL* could be used in breeding. Due to the availability of high-density SNP markers, it is more likely that these QTL represent actual candidate genes for the various traits, as previously identified for pre-harvest sprouting and yellow pigment content (Cabral et al., 2014; Colasuonno et al., 2014). If so they are potential candidates for fine mapping and ultimate candidate gene discovery.

## Conclusion

A high-density linkage map was constructed in the Zhou 8425B/Chinese Spring population using the 90K SNP array; it proved powerful for mapping QTL for yield components, PH and yield-related physiological traits in wheat. Ten pleiotropic QTL clusters for yield related traits and eight novel QTL for TKW, PH, KNS (2), SN (2), and Chl-10 (2) were identified, with genetic distances of 0–1.5 cM from the closest linked SNP markers; therefore, these QTL could serve as target regions for fine mapping, candidate gene discovery, and MAS in wheat breeding.

## Author contributions

FG carried out the experiment and wrote the paper. WW, JL, and AR performed SNP genotyping and data analysis. GY participated in field trials. XX, XW, and ZH designed the experiment and wrote the paper. All authors read and approved the final manuscript.

### Conflict of interest statement

The authors declare that the research was conducted in the absence of any commercial or financial relationships that could be construed as a potential conflict of interest.

## Supplementary material

Table S1 **Summary of means, maxima, minima, and standard deviations for yield components, plant height, and yield-related physiological traits measured in the Zhou 8425B/Chinese Spring population**.

Table S2 **Summary of the genetic map constructed with 246 RILs derived from the Zhou 8425B/Chinese Spring cross**.

Table S3 **All SNP markers mapped in the Zhou 8425B/Chinese Spring populationin**.

Table S4 **The names and positions of 866 newly mapped SNP markers**.

Figure S1 **Frequency distributions of yield components, plant height, and yield-related physiological traits in Zhou 8425B/Chinese Spring population**. (a) Thousand kernel weight, (b) Kernel number per spike, (c) Spike number/m^2^, (d) Plant height, (e) spike length, (f) SPAD value of chlorophyll content at anthesis, (g) SPAD value of chlorophyll content at 10 days post-anthesis, (h): Normalized difference in vegetation index at anthesis, (i) Normalized difference in vegetation index at 10 days post-anthesis. 2012–2013ZK, 2012–2013 cropping season in Zhoukou; 2012–2013ZZ, 2012–2013 cropping season in Zhengzhou; 2013–2014ZK, 2013–2014 cropping season in Zhoukou; 2013–2014ZZ, 2013–2014 cropping season in Zhengzhou.

## Abbreviations

CT: canopy temperature
Chl-A: SPAD value of chlorophyll content at anthesis
Chl-10: SPAD value of chlorophyll content at 10 days post-anthesis
GY: grain yield
GWAS: genome-wide association study
KNS: kernel number per spike
MAS: marker-assisted selection
NDVI-A: normalized difference in vegetation index at anthesis
NDVI-10: normalized difference in vegetation index at 10 days post-anthesis
PH: plant height
QTL: quantitative trait locus/loci
RIL: recombinant inbred line
SN: spike number/m^2^
SL: spike length
SNP: single nucleotide polymorphism
SSR: simple sequence repeat
TKW: thousand kernel weight.

## References

[B1] Akhunov E. Nicolet C. Dvorak J. (2009). Single nucleotide polymorphism genotyping in polyploid wheat with the Illumina Golden-Gate assay. Theor. Appl. Genet. 119, 507–517. 10.1007/s00122-009-1059-5 19449174 PMC2715469

[B2] Ali M. L. Baenziger P. S. Ajlouni Z. A. Campbell B. T. Gill K. S. Eskridge K. M. (2011). Mapping QTL for agronomic traits on wheat chromosome 3A and a comparison of recombinant inbred chromosome line populations. Crop Sci. 51, 553–566. 10.2135/cropsci2010.06.0359

[B3] Aranzana M. J. Kim S. Zhao K. Y. Bakker E. Horton M. Jakob K.. (2005). Genome-wide association mapping in Arabidopsis identifies previously known flowering time and pathogen resistance genes. PLoS Genet. 1:e60. 10.1371/journal.pgen.0010060 16292355 PMC1283159

[B4] Ariyadasa R. Mascherm M. Nussbaumer T. Schulte D. Frenkel Z. Poursarebani N.. (2014). A sequence-ready physical map of barley anchored genetically by two million single-nucleotide polymorphisms. Plant Physiol. 164, 412–423. 10.1104/pp.113.228213 24243933 PMC3875818

[B5] Bagge M. Xia X. C. Lübberstedt T. (2007). Functional markers in wheat. Curr. Opin. Plant Biol. 10, 211–216. 10.1016/jpbi200701009 17292659

[B6] Birkhead T. R. Ball A. Stapleym J. Dawson D. Burke T. Slate J. (2010). A comparison of SNPs and microsatellites as linkage mapping markers: lessons from the zebra finch (*Taeniopygia guttata*). BMC Genomics 11:218. 10.1186/1471-2164-11-218 20359323 PMC2864244

[B7] Barker G. L. A. Edwards K. J. (2009). A genome-wide analysis of single nucleotide polymorphism diversity in the world's major cereal crops. Plant Biotechnol. J. 7, 318–325. 10.1111/j.1467-7652.2009.00412.x 19386040

[B8] Bennett D. Reynolds M. Mullan D. Izanloo A. Kuchel H. Langridge P.. (2012). Detection of two major grain yield QTL in bread wheat (*Triticum aestivum* L.) under heat, drought and high yield potential environments. Theor. Appl. Genet. 125, 1473–1485. 10.1007/s00122-012-1927-2 22772727

[B9] Blanco A. Bellomo M. P. Cenci A. De Giovanni C. D'Ovidio R. Iacono E. (1998). A genetic linkage map of durum wheat. Theor. Appl. Genet. 97, 721–728. 10.1007/s001220050948

[B10] Börner A. Schumann E. Fürste A. Cöster H. Leithold B. Röder M. S.. (2002). Mapping of quantitative trait loci determining agronomic important characters in hexapioid wheat (*Triticum aestivum* L.). Theor. Appl. Genet. 105, 921–936. 10.1007/s00122-002-0994-1 12582918

[B11] Cabral A. L. Jordan M. C. McCartney C. A. You F. M. Humphreys D. G. MacLachlan R.. (2014). Identification of candidate genes, regions and markers for pre-harvest sprouting resistance in wheat (*Triticum aestivum* L.). BMC Plant Biol. 14:340. 10.1186/s12870-014-0340-1 25432597 PMC4253633

[B12] Colasuonno P. Gadaleta A. Giancaspro A. Nigro D. Giove S. Incerti O. (2014). Development of a high-density SNP-based linkage map and detection of yellow pigment content QTLs in durum wheat. Mol. Breeding 34, 1563–1578. 10.1007/s11032-014-0183-3

[B13] Cook J. P. McMullen M. D. Holland J. B. Tian F. Bradbury P. Ross-Ibarra J.. (2012). Genetic architecture of maize kernel composition in the nested association mapping and inbred association panels. Plant Physiol. 158, 824–834. 10.1104/pp.111.185033 22135431 PMC3271770

[B14] Cuthbert J. L. Somers D. J. Brûlé-Babel A. L. Brown P. D. Crow G. H. (2008). Molecular mapping of quantitative trait loci for yield and yield components in spring wheat (*Triticum aestivum* L.). Theor. Appl. Genet. 117, 595–608. 10.1007/s00122-008-0804-5 18516583

[B15] Edae E. A. Byrne P. F. Haley S. D. Lopes M. S. Reynolds M. P. (2014). Genome-wide association mapping of yield and yield components of spring wheat under contrasting moisture regimes. Theor. Appl. Genet. 127, 791–807. 10.1007/s00122-013-2257-8 24408378

[B16] Ellis M. H. Rebetzke G. J. Azanza F. Richards R. A. Spielmeyer W. (2005). Molecular mapping of gibberellin-responsive dwarfing genes in bread wheat. Theor. Appl. Genet. 111, 423–430. 10.1007/s00122-005-2008-6 15968526

[B17] Golabadi M. Arzani A. Mirmohammadi Maibody S. A. M. Tabatabaei B. E. S. Mohammadi S. A. (2011). Identification of microsatellite markers linked with yield components under drought stress at terminal growth stages in durum wheat. Euphytica 177, 207–221. 10.1007/s10681-010-0242-8

[B18] Green A. J. Berger G. Griffey C. A. Pitman R. Thomason W. Balota M. (2012). Genetic yield improvement in soft red winter wheat in the Eastern United States from 1919 to 2009. Crop Sci. 52, 2097–2108. 10.2135/cropsci2012010026

[B19] Gupta P. K. Varshney R. K. Sharma P. C. Ramesh B. (1999). Molecular markers and their applications in wheat breeding. Plant Breeding 118, 369–390. 10.1046/j1439-0523199900401x

[B20] Holland J. B. (2007). Genetic architecture of complex traits in plants. Curr. Opin. Plant Biol. 10, 156–161. 10.1016/jpbi200701003 17291822

[B21] Huang X. Wei X. Sang T. Zhao Q. Feng Q. Zhao Y.. (2010). Genome-wide association studies of 14 agronomic traits in rice landraces. Nat. Genet. 42, 961–967. 10.1038/ng.695 20972439

[B22] Huang X. Zhao Y. Wei X. Li C. Wang A. Zhao Q.. (2011). Genome-wide association study of flowering time and grain yield traits in a worldwide collection of rice germplasm. Nat. Genet. 44, 32–39. 10.1038/ng.1018 22138690

[B23] Huang X. Q. Kempf H. Ganal M. W. Röder M. S. (2004). Advanced backcross QTL analysis in progenies derived from a cross between a German elite winter wheat variety and a synthetic wheat (*Triticum aestivum* L.). Theor. Appl. Genet. 109, 933–943. 10.1007/s00122-004-1708-7 15243706

[B24] Jia G. Huang X. Zhi H. Zhao Y. Zhao Q. Li W.. (2013). A haplotype map of genomic variations and genome-wide association studies of agronomic traits in foxtail millet (*Setaria italica*). Nat. Genet. 45, 957–961. 10.1038/ng.2673 23793027

[B25] Kumar N. Kulwal P. L. Balyan H. S. Gupta P. K. (2007). QTL mapping for yield and yield contributing traits in two mapping populations of bread wheat. Mol. Breeding 19, 163–177. 10.1007/s11032-006-9056-8

[B26] Lambel S. Lanini B. Vivoda E. Fauve J. Wechter W. P. Harris-Shultz K. R.. (2014). A major QTL associated with *Fusarium oxysporum* race resistance identified in genetic populations derived from closely related watermelon lines using selective genotyping and genotyping-by-sequencing for SNP discovery. Theor. Appl. Genet. 127, 2105–2115. 10.1007/s00122-014-2363-2 25104326

[B27] Li H. Peng Z. Yang X. Wang W. Fu J. Wang J.. (2012). Genome-wide association study dissects the genetic architecture of oil biosynthesis in maize kernels. Nat. Genet. 45, 43–50. 10.1038/ng.2484 23242369

[B28] Li H. H. Ye G. Y. Wang J. K. (2007a). A modified algorithm for the improvement of composite interval mapping. Genetics 175, 361–374. 10.1534/genetics.106.06681 17110476 PMC1775001

[B29] Li S. S. Jia J. Z. Wei X. Y. Zhang X. C. Li L. Z. Chen H. M. (2007b). An intervarietal genetic map and QTL analysis for yield traits in wheat. Mol. Breeding 20, 167–178. 10.1007/s11032-007-9080-3

[B30] Li X. M. Xia X. C. Xiao Y. G. He Z. H. Wang D. S. Trethowan R. (2015). QTL mapping for plant height and yield components in common wheat under water limited and full irrigation environments. Crop Pasture Sci. 67, 660–670. 10.1071/CP14236

[B31] Li Z. F. Zheng T. C. He Z. H. Li G. Q. Xu S. C. Li X. P.. (2006). Molecular tagging of stripe rust resistance gene *YrZH84* in Chinese wheat line Zhou 8425B. Theor. Appl. Genet. 112, 1098–1103. 10.1007/s00122-006-0211-8 16450183

[B32] Liu G. Li J. J. Lu L. H. Qin D. D. Zhang J. P. Guan P. F.. (2014). Mapping QTLs of yield-related traits using RIL population derived from common wheat and Tibetan semi-wild wheat. Theor. Appl. Genet. 127, 2415–2432. 10.1007/s00122-014-2387-7 25208643

[B33] Lopes M. S. Reynolds M. P. McIntyre L. Mathews K. L. Jalal Kamali M. R. Mossad M.. (2013). QTL for yield and associated traits in the Seri/Babax population grown across several environments in Mexico, in the West Asia, North Africa, and South Asia regions. Theor. Appl. Genet. 126, 971–984. 10.1007/s00122-012-2030-4 23269228

[B34] Lupton F. H. (1966). Translocation of photosynthetic assimilates in wheat. Ann. Appl. Biol. 57, 355–364. 10.1111/j.1744-7348.1966.tb03829.x

[B35] Marone D. Laidò G. Gadaleta A. Colasuonno P. Ficco D. B. M. Giancaspro A.. (2012). A high-density consensus map of A and B wheat genomes. Theor. Appl. Genet. 125, 1619–1638. 10.1007/s00122-012-1939-y 22872151 PMC3493672

[B36] McCartney C. A. Somers D. J. Humphreys D. J. Lukow O. (2005). Mapping quantitative trait loci controlling agronomic traits in the spring wheat cross RL 4452 × AC ‘Domain’. Genome 48, 870–883. 10.1139/g05-055 16391693

[B37] Nachit M. M. Elouafi I. Pagnotta M. A. Ei S. A. Iacono E. Labhilili M. (2001). Molecular linkage map for an intraspecific recombinant inbred population of durum wheat (*Triticum turgidum* L. var. *durum*). Theor. Appl. Genet. 102, 177–186. 10.1007/s001220051633

[B38] Peng Z. S. Li X. Yang Z. J. Liao M. L. (2011). A new reduced height gene found in the tetraploid semi-dwarf wheat landrace Aiganfanmai. Genet. Mol. Res. 10, 2349–2357. 10.4238/2011.October.5.5 22002128

[B39] Pinto R. S. Reynolds M. P. (2015). Common genetic basis for canopy temperature depression under heat and drought stress associated with optimized root distribution in bread wheat. Theor. Appl. Genet. 128, 575–585. 10.1007/s00122-015-2453-9 25707766 PMC4361760

[B40] Prashar A. Hornyik C. Young V. McLean K. Sharma S. K. Dale M. F. B.. (2014). Construction of a dense SNP map of a highly heterozygous diploid potato population and QTL analysis of tuber shape and eye depth. Theor. Appl. Genet. 127, 2159–2171. 10.1007/s00122-014-2369-9 25159608

[B41] Quarrie S. A. Quarrie S. P. Radosevic R. Rancic D. Kaminska A. Barnes J. D.. (2006). Dissecting a wheat QTL for yield present in a range of environments: from the QTL to candidate genes. J. Exp. Bot. 57, 2627–2637. 10.1093/jxb/erl026 16831847

[B42] Rafalski A. (2002). Applications of single nucleotide polymorphisms in crop genetics. Curr. Opin. Plant Biol. 5, 94–100. 10.1016/S1369-5266(02)00240-6 11856602

[B43] Rebetzke G. J. Condon A. G. Farquhar G. D. Appels R. Richards R. A. (2008a). Quantitative trait loci for carbon isotope discrimination are repeatable across environments and wheat mapping populations. Theor. Appl. Genet. 118, 123–137. 10.1007/s00122-008-0882-4 18818897

[B44] Rebetzke G. J. van Herwaarden A. F. Jenkins C. Weiss M. Lewis D. Ruuska S. (2008b). Quantitative trait loci for water-soluble carbohydrates and associations with agronomic traits in wheat. Aust. J. Agric. Res. 59, 891–905. 10.1071/AR08067

[B45] Reynolds M. Bonnett D. Chapman S. C. Furbank R. T. Manes Y. Mather D. E.. (2011). Raising yield potential of wheat. I. Overview of a consortium approach and breeding strategies. J. Exp. Bot. 62, 439–452. 10.1093/jxb/erq311 20952629

[B46] Reynolds M. Tuberosam R. (2008). Translational research impacting on crop productivity in drought-prone environments. Curr. Opin. Plant Biol. 11, 171–179. 10.1016/j.pbi.2008.02.005 18329330

[B47] Schlotterer C. (2004). The evolution of molecular markers - just a matter of fashion. Nat. Rev. Genet. 5, 63–69. 10.1038/nrg1249 14666112

[B48] Sela H. Ezratim S. Ben-Yehuda P. Manisterski J. Akhunov E. Dvorak J.. (2014). Linkage disequilibrium and association analysis of stripe rust resistance in wild emmer wheat (*Triticum turgidum* ssp. *dicoccoides)* population in Israel. Theor. Appl. Genet. 127, 2453–2463. 10.1007/s00122-014-2389-5 25223542

[B49] Shiaoman C. Zhang W. J. Akhunov E. Sherman J. Ma Y. Q. Luo M. C. (2009). Analysis of gene-derived SNP marker polymorphism in US wheat cultivars. Mol. Breeding 23, 23–33. 10.1007/s11032-008-9210-6

[B50] Sindhu A. Ramsay L. Sanderson L. A. Stonehouse R. Li R. Condie J.. (2014). Gene-based SNP discovery and genetic mapping in pea. Theor. Appl. Genet. 127, 2225–2241. 10.1007/s00122-014-2375-y 25119872 PMC4180032

[B51] Song Q. Hyten D. L. Jia G. Quigley C. V. Fickus E. W. Nelson R. L.. (2013). Development and evaluation of Soy SNP 50K, a high-density genotyping array for soybean. PLoS ONE 8:e54985. 10.1371/journal.pone.0054985 23372807 PMC3555945

[B52] Stam P. (1993). Construction of integrated genetic linkage maps by means of a new computer package: JoinMap. Plant J. 3, 739–744. 10.1111/j.1365-313X.1993.00739.x

[B53] Sukumaran S. Dreisigacker S. Lopes M. Chavez P. Reynolds M. P. (2015). Genome-wide association study for grain yield and related traits in an elite spring wheat population grown in temperate irrigated environments. Theor. Appl. Genet. 128, 353–363. 10.1007/s00122-014-2435-3 25490985

[B54] Tian F. Bradbury P. J. Brown P. J. Hung H. Sun Q. Flint-Garcia S.. (2011). Genome-wide association study of leaf architecture in the maize nested association mapping population. Nat. Genet. 43, 159–162. 10.1038/ng.746 21217756

[B55] Wang S. C. Wong D. Forrest K. Allen A. Chao S. M. Huang B. E.. (2014). Characterization of polyploid wheat genomic diversity using a high-density 90000 single nucleotide polymorphism array. Plant Biotechnol. J. 12, 87–96. 10.1111/pbi.12183 24646323 PMC4265271

[B56] Xiao Y. G. Yin G. H. Li H. H. Xia X. C. Yan J. Zheng T. C. (2011). Genetic diversity and genome-wide association analysis of stripe rust resistance among the core wheat parent Zhou 8425B and its derivatives. Sci. Agric. Sin. 44, 3919–3929. 10.3864/j.issn.0578-1752.2011.19.001

[B57] Xu Y. F. An D. G. Liu D. C. Zhang A. M. Xu H. X. Li B. (2012). Mapping QTLs with epistatic effects and QTL × treatment interactions for salt tolerance at stage of wheat. Euphytica 186, 233–245. 10.1007/s10681-012-0647-7

[B58] Yang Q. Li Z. Li W. Q. Ku L. X. Wang C. Ye J. R.. (2013). CACTA-like transposable element in *ZmCCT* attenuated photoperiod sensitivity and accelerated the postdomestication spread of maize. Proc. Natl. Acad. Sci. U.S.A. 110, 16969–16974. 10.1073/pnas.1310949110 24089449 PMC3801022

[B59] Yang Z. B. Bai Z. Y. Li X. L. Wang P. Wu Q. X. Yang L.. (2012). SNP identification and allelic-specific PCR markers development for *TaGW2*, a gene linked to wheat kernel weight. Theor. Appl. Genet. 125, 1057–1068. 10.1007/s00122-012-1895-6 22643902

[B60] Yin G. H. Wang J. W. Wen W. E. He Z. H. Li Z. F. Wang H. (2009). Mapping of wheat stripe rust resistance gene *YrZH84* with RGAP markers and its application. Acta Agron. Sin. 35, 1274–1281. 10.3724/SP.J.1006.2009.01274

[B61] Yu H. Xie W. Wang J. Xing Y. Xu C. Li X.. (2011). Gains in QTL detection using an ultra-high density SNP map based on population sequencing relative to traditional RFLP/SSR markers. PLoS ONE 6:e17595. 10.1371/journal.pone.0017595 21390234 PMC3048400

[B62] Zhao K. Tung C. W. Eizenga G. C. Wright M. H. Ali M. L. Price A. H.. (2011). Genome-wide association mapping reveals a rich genetic architecture of complex traits in *Oryza sativa*. Nat. Commun. 2:467. 10.1038/ncomms1467 21915109 PMC3195253

[B63] Zhao X. L. Zheng T. C. Xia X. C. He Z. H. Liu D. Q. Yang W. X.. (2008). Molecular mapping of leaf rust resistance gene *LrZH84* in Chinese wheat line Zhou 8425B. Theor. Appl. Genet. 117, 1069–1075. 10.1007/s00122-008-0845-9 18651124

