# Supplementary material for: Genome-Wide Linkage Mapping of QTL for Yield Components, Plant Height and Yield-Related Physiological Traits in the Chinese Wheat Cross Zhou 8425B/Chinese Spring
Source: Front Plant Sci. 2015 Dec 18;6:1099. doi: 10.3389/fpls.2015.01099 (PMC4683206; doi:10.3389/fpls.2015.01099)
Supplement: Table S1 — Summary of means, maxima, minima, and standard deviations for yield components, plant height, and yield-related physiological traits measured in the Zhou 8425B/Chinese Spring population. [file Table1.docx]

Table S1 Summary of means, maxima, minima and standard deviations for yield components, plant height and yield-related physiological traits measured in the Zhou 8425B/Chinese Spring population

| Trait | Zhou 8425B | Chinese Spring | Mean | Range |
| --- | --- | --- | --- | --- |
| TKW (g) | 52.6 | 29.1 | 37.2±4.4 | 26.5-52.6 |
| KNS | 49 | 56 | 53±6 | 41-74 |
| SN | 426 | 588 | 465±66 | 318-671 |
| SL (cm) | 11.5 | 8.3 | 10.2±1.2 | 6.9-16.0 |
| PH (cm) | 67.1 | 115.1 | 100.9±14.3 | 60.6-125.9 |
| Chl-A | 49.1 | 42.8 | 46.5±3.2 | 38.6-76.5 |
| Chl-10 | 55.5 | 46.4 | 47.8±4.0 | 28.7-58.1 |
| NDVI-A | 0.75 | 0.74 | 0.74±0.03 | 0.51-0.81 |
| NDVI-10 | 0.62 | 0.60 | 0.55±0.06 | 0.40-0.71 |

TKW: thousand kernel weight; PH: plant height; SL: spike length; KNS: kernel number per spike; SN: spike number/m^2^; Chl-A: SPAD value of chlorophyll content at anthesis; Chl-10: SPAD value of chlorophyll content at 10 days post-anthesis; NDVI-A: normalized difference in vegetation index at anthesis; NDVI-10: normalized difference in vegetation index at 10 days post-anthesis
