# Supplementary material for: Genome-Wide Linkage Mapping of QTL for Yield Components, Plant Height and Yield-Related Physiological Traits in the Chinese Wheat Cross Zhou 8425B/Chinese Spring
Source: Front Plant Sci. 2015 Dec 18;6:1099. doi: 10.3389/fpls.2015.01099 (PMC4683206; doi:10.3389/fpls.2015.01099)
Supplement: Table S2 — Summary of the genetic map constructed with 246 RILs derived from the Zhou 8425B/Chinese Spring cross. [file Table2.DOCX]

Table S2 Summary of the genetic map constructed with 246 RILs derived from the Zhou 8425B/Chinese Spring cross

| Chromosome | No. of  markers | Map length  (cM) | Marker density  (cM/marker) |
| --- | --- | --- | --- |
| 1A | 238 | 182.0 | 0.76 |
| 1B | 417 | 171.8 | 0.41 |
| 1D | 106 | 104.1 | 0.98 |
| 2A | 360 | 269.0 | 0.75 |
| 2B | 516 | 145.2 | 0.28 |
| 2D | 98 | 144.6 | 1.48 |
| 3A | 297 | 238.0 | 0.80 |
| 3B | 425 | 200.4 | 0.47 |
| 3D | 10 | 91.0 | 9.10 |
| 4A | 262 | 179.8 | 0.69 |
| 4B | 155 | 120.4 | 0.78 |
| 4D | 22 | 145.5 | 6.61 |
| 5A | 378 | 271.3 | 0.72 |
| 5B | 599 | 223.6 | 0.37 |
| 5D | 55 | 40.9 | 0.74 |
| 6A | 445 | 224.3 | 0.50 |
| 6B | 347 | 190.7 | 0.55 |
| 6D | 34 | 21.0 | 0.62 |
| 7A | 477 | 303.7 | 0.64 |
| 7B | 379 | 224.7 | 0.59 |
| 7D | 16 | 117.4 | 7.34 |
| Hom. group 1 | 761 | 457.9 | 0.60 |
| Group 2 | 974 | 558.8 | 0.57 |
| Group 3 | 732 | 529.4 | 0.72 |
| Group 4 | 439 | 445.7 | 1.02 |
| Group 5 | 1032 | 535.8 | 0.52 |
| Group 6 | 826 | 436 | 0.53 |
| Group 7 | 872 | 645.8 | 0.74 |
| A genome | 2457 | 1668.1 | 0.68 |
| B genome | 2838 | 1276.8 | 0.45 |
| D genome | 341 | 664.5 | 1.95 |
| Total | 5636 | 3609.4 | 0.64 |
