# Supplementary figures and images for: Genome-Wide Linkage Mapping of QTL for Yield Components, Plant Height and Yield-Related Physiological Traits in the Chinese Wheat Cross Zhou 8425B/Chinese Spring
Source: Front Plant Sci. 2015 Dec 18;6:1099. doi: 10.3389/fpls.2015.01099 (PMC4683206; doi:10.3389/fpls.2015.01099)

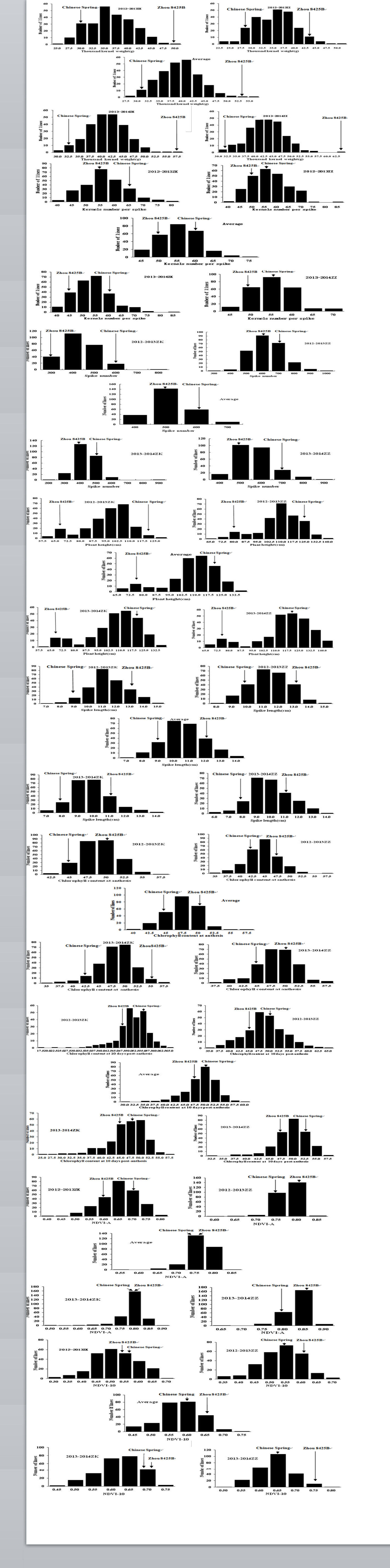

Supplement: Figure S1 — Frequency distributions of yield components, plant height, and yield-related physiological traits in Zhou 8425B/Chinese Spring population. (a) Thousand kernel weight, (b) Kernel number per spike, (c) Spike number/m2, (d) Plant height, (e) spike length, (f) SPAD value of chlorophyll content at anthesis, (g) SPAD value of chlorophyll content at 10 days post-anthesis, (h): Normalized difference in vegetation index at anthesis, (i) Normalized difference in vegetation index at 10 days post-anthesis. 2012–2013ZK, 2012–2013 cropping season in Zhoukou; 2012–2013ZZ, 2012–2013 cropping season in Zhengzhou; 2013–2014ZK, 2013–2014 cropping season in Zhoukou; 2013–2014ZZ, 2013–2014 cropping season in Zhengzhou. [file Image1.JPEG]
